# Genome-wide association studies and CRISPR/Cas9-mediated gene editing identify regulatory variants influencing eyebrow thickness in humans

**DOI:** 10.1371/journal.pgen.1007640

**Published:** 2018-09-24

**Authors:** Sijie Wu, Manfei Zhang, Xinzhou Yang, Fuduan Peng, Juan Zhang, Jingze Tan, Yajun Yang, Lina Wang, Yanan Hu, Qianqian Peng, Jinxi Li, Yu Liu, Yaqun Guan, Chen Chen, Merel A. Hamer, Tamar Nijsten, Changqing Zeng, Kaustubh Adhikari, Carla Gallo, Giovanni Poletti, Lavinia Schuler-Faccini, Maria-Cátira Bortolini, Samuel Canizales-Quinteros, Francisco Rothhammer, Gabriel Bedoya, Rolando González-José, Hui Li, Jean Krutmann, Fan Liu, Manfred Kayser, Andres Ruiz-Linares, Kun Tang, Shuhua Xu, Liang Zhang, Li Jin, Sijia Wang

**Affiliations:** 1 CAS Key Laboratory of Computational Biology, CAS-MPG Partner Institute for Computational Biology, Shanghai Institute of Nutrition and Health, Shanghai Institutes for Biological Sciences, University of Chinese Academy of Sciences, Chinese Academy of Sciences, Shanghai, China; 2 State Key Laboratory of Genetic Engineering and Ministry of Education Key Laboratory of Contemporary Anthropology, Collaborative Innovation Center for Genetics and Development, School of Life Sciences, Fudan University, Shanghai, China; 3 Human Phenome Institute, Fudan University, 825 Zhangheng Road, Shanghai, China; 4 CAS Key Laboratory of Tissue Microenvironment and Tumor, Shanghai Institute of Nutrition and Health, Shanghai Institutes for Biological Sciences, University of Chinese Academy of Sciences, Chinese Academy of Sciences, Shanghai, China; 5 SIBS (Institute of Health Sciences) Changzheng Hospital Joint Center for Translational Research, Institutes for Translational Research (CAS-SMMU), Shanghai, China; 6 Key laboratory of Genomic and Precision Medicine, Beijing Institute of Genomics, Chinese Academy of Sciences, Beijing, China; 7 Fudan-Taizhou Institute of Health Sciences, Taizhou, Jiangsu, China; 8 Department of Biochemistry, Preclinical Medicine College, Xinjiang Medical University, Urumqi, China; 9 Department of Stomatology, Chang Zheng Hospital, Second Military Medical University, Shanghai, China; 10 Department of Dermatology, Erasmus MC University Medical Center Rotterdam, CA Rotterdam, The Netherlands; 11 Department of Genetics, Evolution and Environment, and UCL Genetics Institute, University College London, London, United Kingdom; 12 Laboratorios de Investigación y Desarrollo, Facultad de Ciencias y Filosofía, Universidad Peruana Cayetano Heredia, Lima, Peru; 13 Departamento de Genética, Universidade Federal do Rio Grande do Sul, Porto Alegre Brasil; 14 Unidad de Genómica de Poblaciones Aplicada a la Salud, Facultad de Química, UNAM-Instituto Nacional de Medicina Genómica, México City, México; 15 Instituto de Alta Investigación, Universidad de Tarapacá, Arica, Chile; 16 Laboratorio de Genética Molecular (GENMOL), Universidad de Antioquia, Medellín, Colombia; 17 Instituto Patagónico de Ciencias Sociales y Humanas, Centro Nacional Patagónico, CONICET, Puerto Madryn, Argentina; 18 IUF-Leibniz Research Institute for Environmental Medicine, Dusseldorf, Germany; 19 Department of Genetic Identification, Erasmus MC University Medical Center Rotterdam, CA Rotterdam, The Netherlands; 20 School of Life Science and Technology, ShanghaiTech University, Shanghai, China; 21 Center for Excellence in Animal Evolution and Genetics, Chinese Academy of Sciences, Kunming China; HudsonAlpha Institute for Biotechnology, UNITED STATES

## Abstract

Hair plays an important role in primates and is clearly subject to adaptive selection. While humans have lost most facial hair, eyebrows are a notable exception. Eyebrow thickness is heritable and widely believed to be subject to sexual selection. Nevertheless, few genomic studies have explored its genetic basis. Here, we performed a genome-wide scan for eyebrow thickness in 2961 Han Chinese. We identified two new loci of genome-wide significance, at 3q26.33 near *SOX2* (rs1345417: P = 6.51×10^−10^) and at 5q13.2 near *FOXD1* (rs12651896: P = 1.73×10^−8^). We further replicated our findings in the Uyghurs, a population from China characterized by East Asian-European admixture (N = 721), the CANDELA cohort from five Latin American countries (N = 2301), and the Rotterdam Study cohort of Dutch Europeans (N = 4411). A meta-analysis combining the full GWAS results from the three cohorts of full or partial Asian descent (Han Chinese, Uyghur and Latin Americans, N = 5983) highlighted a third signal of genome-wide significance at 2q12.3 (rs1866188: P = 5.81×10^−11^) near *EDAR*. We performed fine-mapping and prioritized four variants for further experimental verification. CRISPR/Cas9-mediated gene editing provided evidence that rs1345417 and rs12651896 affect the transcriptional activity of the nearby *SOX2* and *FOXD1* genes, which are both involved in hair development. Finally, suitable statistical analyses revealed that none of the associated variants showed clear signals of selection in any of the populations tested. Contrary to popular speculation, we found no evidence that eyebrow thickness is subject to strong selective pressure.

## Author summary

Hair plays an important role in primates and is clearly subject to adaptive selection. While humans have lost most facial hair, eyebrows are a notable exception. Eyebrow thickness is heritable and widely believed to be subject to sexual selection. Nevertheless, few genomic studies have explored its genetic basis. Here we performed genome-wide association studies for eyebrow thickness in multiple ethnic groups, including Han Chinese, Uyghurs, Latin Americans, and Caucasians. We found solid evidence that novel genetic variants near the *SOX2*, *FOXD1* and *EDAR* genes could affect eyebrow thickness. After fine mapping, we prioritized four variants for experimental verification. CRISPR/Cas9-mediated gene editing provided evidence that the variants rs1345417 and rs12651896 affect the transcriptional activity of the nearby *SOX2* and *FOXD1* genes. This represents a successful example of a combination of GWAS and CRISPR/Cas9 technology to demonstrate how non-coding variants with regulatory functions may play an important role in common diseases and traits. Finally, suitable statistical analyses suggest that, contrary to popular speculation, eyebrow thickness should not be subject to strong selection pressure, including sexual selection.

## Introduction

Hair has a range of important functions in primates and has been speculated to be subject to intense natural and sexual selection [1]. Although humans have lost most terminal body hair to allow the development of more efficient sweating as an adaptation to bipedal life, considerable facial hair remains, with great diversity between populations [2]. The eyebrow, an area of thick, short facial hair above the eye that follows the shape of the lower margin of the brow ridge, is one of the most conspicuous features of the face. It is thought that its main function is to prevent sweat, water, and other debris from getting into the eye [2]. As a major facial feature, the eyebrow plays an important role in human communication, facial expression, sexual dimorphism and attractiveness [3–6]. It has been suggested that eyebrows may be subject to sexual selection [7]. Notably, eyebrow thickness varies between and within human populations [8,9]. A recent genome-wide association study in Latin Americans found that common DNA variants in the *FOXL2* gene are associated with eyebrow thickness. However, these variants have very low minor allele frequencies in East Asians and Europeans, suggesting that in these two populations, eyebrow thickness may well be affected by different genes.

To enhance our understanding of the genetic basis underlying the variation of human eyebrow thickness, we conducted a genome-wide association study in East Asians and Eurasians, followed by a trans-ethnic meta-analysis, to identify genetic variants that affect eyebrow thickness in humans. Moreover, in order to validate the findings from our association analyses, we performed fine mapping and conducted functional genetic experiments. Finally, we applied various statistical genetics methods to search for signals of positive selection in and around the identified candidate genes.

## Results

### Genome-wide association analysis identified three novel loci associated with eyebrow thickness

**Fig 1:**
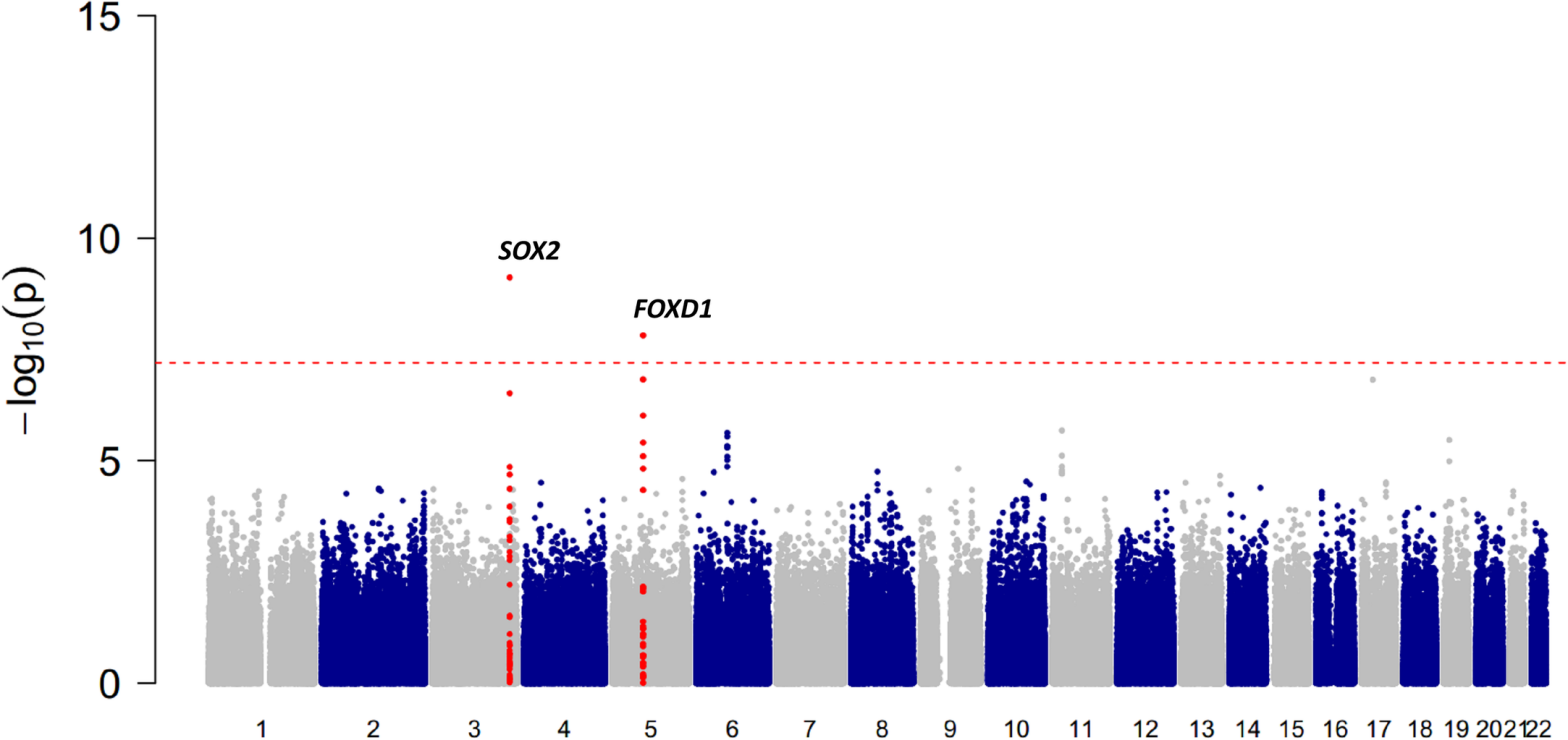
Manhattan plot showing the results of the GWAS for eyebrow thickness in Han Chinese. Manhattan plot illustrating the results of the genome-wide scan for eyebrow thickness in 2961 Han Chinese after adjusting for the top four PCs, gender and age. The red line indicates the threshold for genome-wide statistical significance (*P*<5×10^−8^). Red dots represent SNPs that are close (<5 kb) to the genome-wide significant signals.

Our discovery GWAS included a total of 2961 subjects from the Taizhou Longitudinal Study [10] (TZL, Han Chinese). Replication cohorts were drawn from the Xinjiang Uyghur Study [11] (UYG, N = 721, Uyghur, an admixed East Asian-European population), the CANDELA study [8] (CANDELA, N = 2301, Latin Americans) and the Rotterdam Study [12,13] (RS, N = 4411, Northwestern Europeans; for sample characteristics see S1 Table). In all cohorts, eyebrow thickness was assessed on an ordered categorical scale using a self-developed photo numeric approach (S1 Fig). Inter-rater (for the CANDELA cohort, intra-rater) reliability was reasonable for all cohorts (Kappa: 0.48–0.66, S2 Table). There was a higher degree of diversity in eyebrow thickness in Uyghurs (D = 0.58, Gini-index) and Northern Europeans (D = 0.57) than in Han Chinese (D = 0.52) and Latin Americans (D = 0.49) (S1 Table). Eyebrows were significantly less thick in females than in males in TZL, UYG, and RS (*P*_TZL_ = 7.23×10^−70^, *P*_UYG_ = 7.31×10^−33^, *P*_RS_ = 4.10×10^−106^; the CANDELA sample included only male subjects). Age was negatively correlated with eyebrow thickness in TZL, CANDELA, and RS (*P*_TZL_ = 7.75×10^−20^, *P*_CANDELA_ = 4.20×10^−17^, *P*_RS_ = 1.33×10^−10^; the UYG sample included only young subjects between 18 and 24 years). The GWAS in Han Chinese was based on 7,119,456 SNPs; it identified association signals of genome-wide significance (*P*<5×10^−8^) for two genomic regions, located at 3q26.33 (rs1345417: *P* = 6.51×10^−10^) and 5q13.2 (rs12651896: *P* = 1.73×10^−8^; Fig 1). After conditioning on the genotypes of rs1345417 and rs12651896, no additional SNPs showed significant association at a genome-wide level (*P*<5×10^−8^). The heritability of eyebrow thickness in TZL was 37.6%. The SNPs rs1345417 and rs12651896 explain 3.11% and 2.55% of this heritability, respectively. Although the GWAS in the Uyghurs, which was based on 7,224,952 SNPs, did not detect any genome-wide significant signals (S2 Fig), the signals at 3q26.33 and 5q13.2 showed marginal significance (rs1345417: *P* = 3.78×10^−4^; rs12651896: *P* = 5.4×10^−2^), and allelic effects were consistent with those in TZL (Table 1).

**Table 1:** GWAS results for lead SNPs significantly associated with eyebrow thickness.

SNP	Chr.	Position^a^	Gene	Allele	Population	Freq.	Beta	P value
rs1866188	2	109257152	EDAR (253kb up)	A	TZL	0.92	0.096	1.46×10^−4^
					UYG	0.35	0.09	3.42×10^−3^
					CANDELA	0.39	0.087	3.54×10^−6^
					RS	0.06	0.064	0.0141
rs112458845	3	138675741	FOXL2	G	TZL	0.065	-0.009	0.729
					UYG	0.032	0.056	0.494
					CANDELA	0.27	-0.127	4.95×10^−11^
					RS	0.00	0.089	0.366
rs1345417	3	181511951	SOX2 (79kb down)	G	TZL	0.27	0.092	6.51×10^−10^
					UYG	0.54	0.105	3.78×10^−4^
					CANDELA	0.52	0.098	1.04×10^−7^
					RS	0.58	0.04	4.03×10^−3^
rs12651896	5	72502029	FOXD1 (242kb down)	C	TZL	0.27	0.084	1.73×10^−8^
					UYG	0.26	0.064	0.054
					CANDELA	0.32	0.08	7.54×10^−6^
					RS	0.71	0.05	2.93×10^−4^

^a^Position according to human reference NCBI37/hg19.

**Fig 2:**
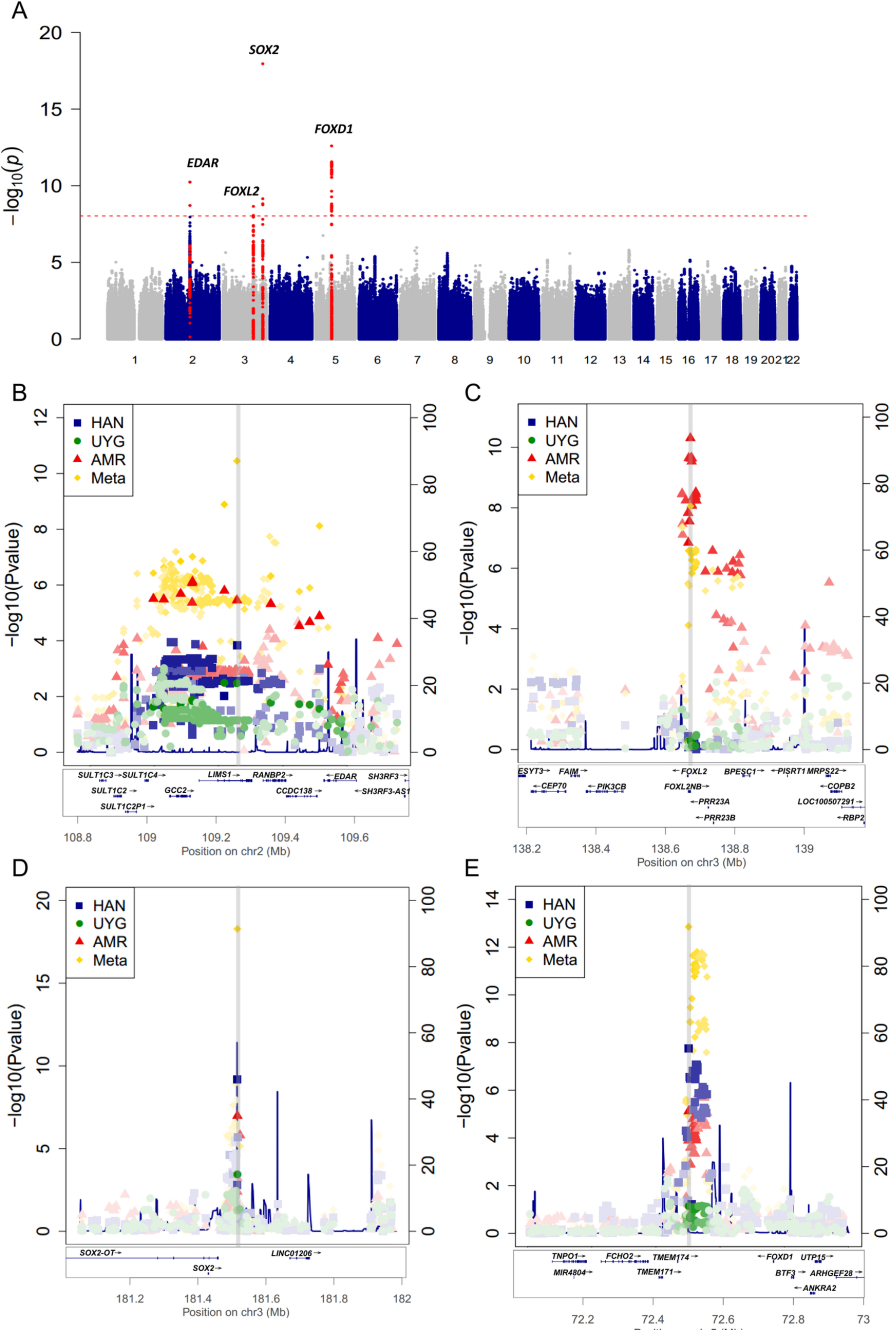
Manhattan plot showing the results of the GWAS meta-analysis for eyebrow thickness in Han Chinese, Uyghurs, and Latin Americans. A) Manhattan plot illustrating results of the transethnic meta-analysis of 2961 Han Chinese, 721 Uyghurs and 2301 Latin Americans. The red line indicates the threshold for genome-wide statistical significance (P<5×10^−8^). Red dots represent SNPs that are close (<5 kb) to the genome-wide significant signals. Regional association and linkage disequilibrium plots are shown for the significantly associated regions around B) rs1866188, C) rs112458845, D) rs1345417 and E) rs12651896 (gray bar). Different colors denote different populations. Increasing color intensities represent an increasing degree of linkage disequilibrium (r^2^) with the top SNP in each panel. The recombination rate (right-hand y axis) is plotted in blue and is based on the ASN population from the 1000 Genome Project. Exons for each gene are represented by vertical bars below the x axis, based on all isoforms available from the hg19 UCSC Genome Browser.

The GWAS in Latin Americans has been published previously; it found that common variants at 3q22.3 are associated with eyebrow thickness [8]. We found that it also corroborated our findings at 3q26.33 and 5q13.2 (rs1345417: *P* = 1.04×10^−7^; rs12651896: *P* = 7.54×10^−6^). The meta-analysis of all three GWAS (TZL, UYG and CANDELA) identified four loci reaching genome-wide significance (*P*<5×10^−8^; Fig 2A; S3 Table). Apart from the loci described above 3q26.33 (rs1345417: *P* = 1.11×10^−19^), 5q13.2 (rs12651896: *P* = 2.52×10^−13^) and 3q22.3 (rs112458845: *P* = 2.24×10^−9^) there was one additional locus of genome-wide significance, at 2q12.3 (rs1866188: *P* = 5.81×10^−11^) (Fig 2). The respective quantile-quantile (Q-Q) plots showed no sign of inflation for any of the association tests described above, and the genomic control factor λ was relatively low in all cases (λ_TZL_ = 1.041, λ_UYG_ = 1.053, λ_CANDELA_ = 1.015, λ_Meta_ = 1.040, S3 Fig), indicating that the test statistics were not substantially confounded by population sub-stratification.

For all three novel loci (3q26.33, 5q13.2 and 2q12.3), the allelic effects acted in the same direction in all populations (Table 1). There was no significant effect size heterogeneity across populations for any of these three association signals (S3 Table). However, the signal at 3q22.3, which was highly significant in CANDELA, with *P* = 4.95×10^−11^ at rs112458845 and an effect allele frequency (EAF) of 0.127, did not reach nominal significance in our GWAS of TZL (EAF = 0.065, *P* = 0.729) and UYG (EAF = 0.032, *P* = 0.494). Furthermore, our meta-analysis revealed strong allelic heterogeneity for rs112458845 (Q = 16.284, I^2^ = 81.58%; S3 Table), indicating that the effect at 3q22.3 may be specific to Latin Americans.

We performed a further replication analysis for all SNPs described above in the Rotterdam Study sample, which comprises 4411 Dutch Europeans. With the exception of rs112458845 at 3q22.3, which was specific to Latin Americans, this analysis replicated all signals at a nominal significance level (rs1866188: *P* = 0.0141; rs112458845: *P* = 0.366; rs1345417: *P* = 4.03×10^−3^; rs12651896: *P* = 2.93×10^−4^; Table 1). The allelic effects at all associated SNPs acted in the same direction as in all other populations tested (Table 1).

### Fine mapping of associated loci suggests four causal variants affecting eyebrow thickness

**Fig 3:**
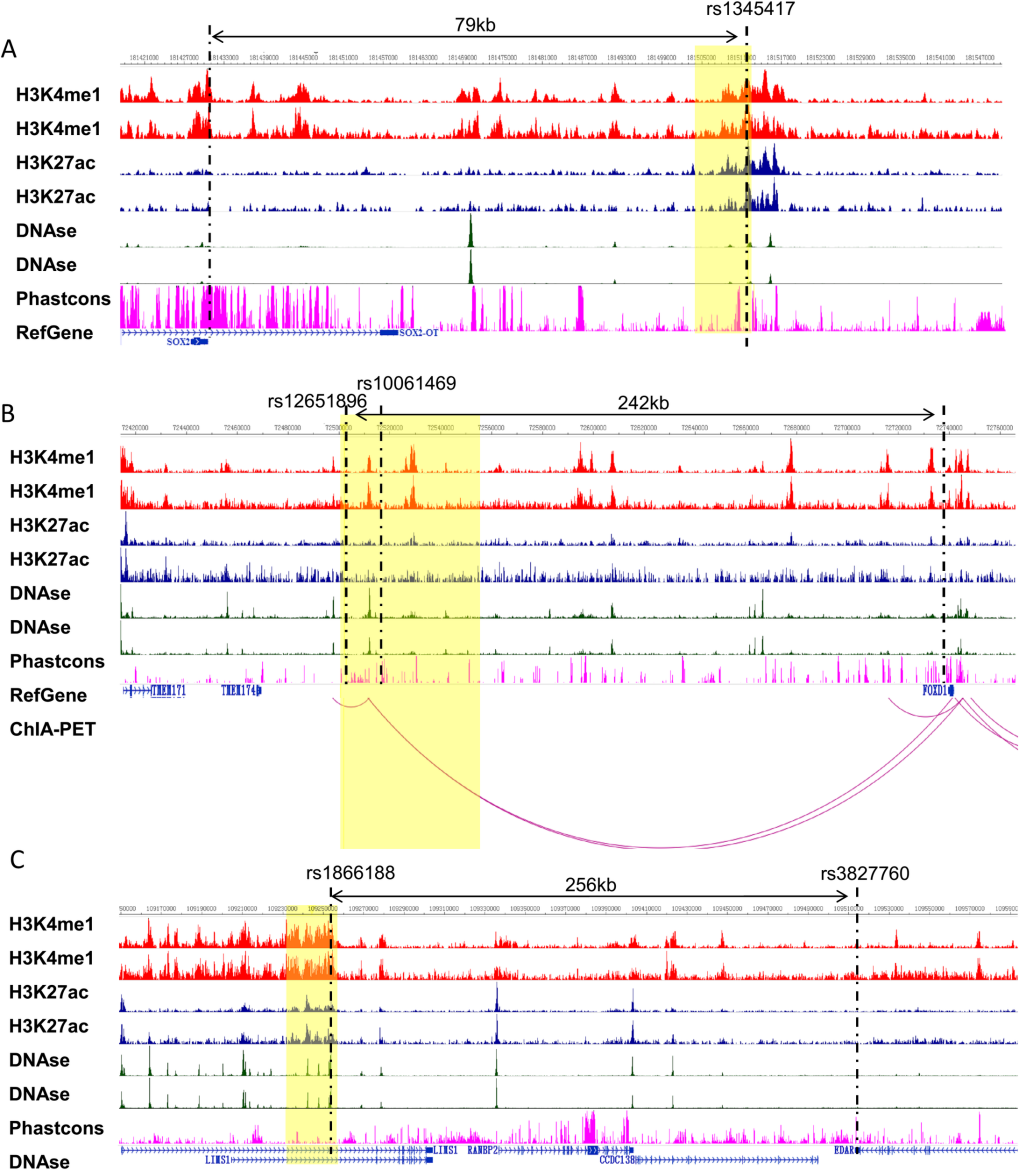
Epigenetic annotation suggests the presence of putative regulatory regions at loci associated with eyebrow thickness. Epigenetic annotation at the significantly associated regions around A) rs1345417 (3q26.33), B) rs12651896 (5q13.2) and C) rs1866188 (2q12.3), based on ENCODE and REMC project data. Regions of significant association (*P*<5×10^−8^) are denoted by a yellow box, with the top signal indicated by a black dashed line. The region exhibits distinct active enhancer signatures defined by epigenetic marks, such as H3K4me1 (red) and H3K27ac (blue) histone modifications and DNase hypersensitivity (green) in foreskin melanocyte primary cells, based on two independent biological replicates. Phastcons (pink) indicates the evolutionary conservation. ChIA-PET indicates long-range chromatin interaction. The different tracks were overlaid with physical positions using the WashU Epigenome Browser.

Next, we sought to localize the variants driving the association signals at each of the novel loci that attained genome-wide significance in our trans-ethnic meta-analysis. We utilized PAINTOR [14,15] to calculate the Bayesian posterior causal probability for each variant included in the signal and identified the sets of variants that collectively explained 99% of the total probability (credible sets). The credible sets for 2q12.3 and 3q26.33 contained a single SNP each (S4 Table). The credible set for 5q13.2 comprised nine variants. Among these, the posterior causal probability was highest for rs12651896 (posterior probability = 0.744; S4 Table). Using CADD [16] and DeepSEA [17] to assess the functional consequences of each variant, we found that rs10061469 was consistently predicted to be functionally important (S4 Table). Our interrogation of ENCODE [18] and REMC [19] data revealed that the regions around rs1866188 and rs1345417 show distinct active enhancer signatures defined by epigenetic markers, such as the histone modifications H3K4me1 and H3K27ac, and DNase hypersensitivity in epithelial cells (Fig 3). The regions around rs12651896 and rs10061469 are involved in long-range chromatin looping interactions with *FOXD1* (Fig 3B). Together, these data suggest the presence of putative regulatory regions in the neighborhood of these variants. We thus chose rs1345417, rs12651896, rs10061469 and rs1866188 for further experimental verification.

### Validating regulatory activity within regions underlying the association signals

**Fig 4:**
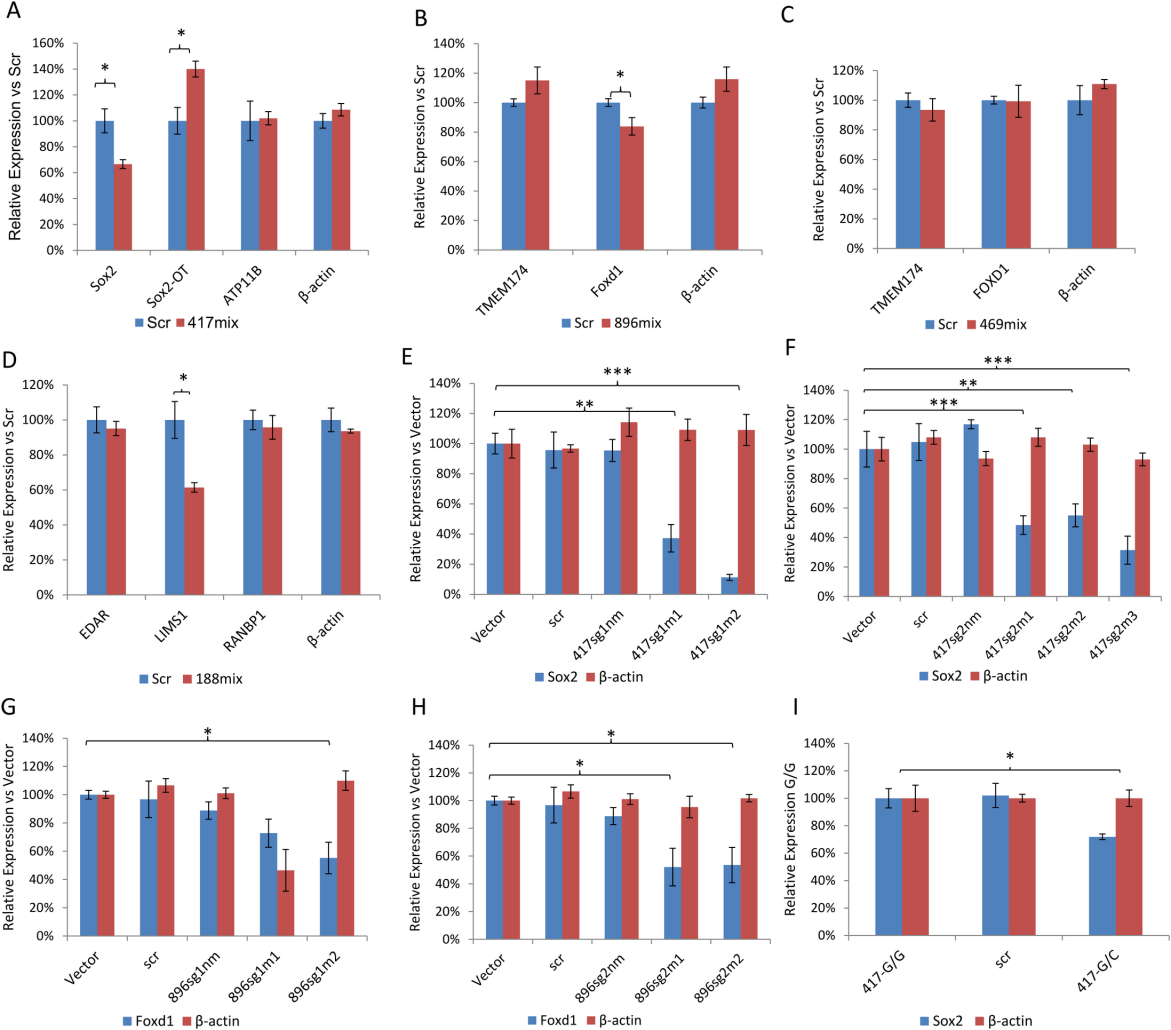
Cas9-mediated gene editing provided evidence that rs1345417 and rs12651896 affect the transcriptional activity of the nearby *SOX2* and *FOXD1*. (A-D) qRT-PCR analysis of relative expression of nearby genes near (A) rs1345417, (B) rs12651896, (C) rs10051469 and (D) rs1866188. Scr denotes lentiCRISPR-Scramble sgRNA infected cells. 417mix, 896mix, 469mix and188mix denote CRISPR-Cas9 edited mixed clones for rs1345417, rs12651896, rs10051469 and rs1866188, respectively. (E-H) qRT-PCR analysis of relative *SOX2* and *FOXD1* expression in the CRISPR-Cas9 edited A375 cell clones. Especially, E) 417sg1m1-2: clone 1 and 2 edited by sgRNA1 for rs1345417; F) 417sg2m1-3: clone 1–3 edited by sgRNA2 for rs1345417. G) 896sg1m1-2: clone 1 and 2 edited by sgRNA1 for rs12651896. H) 896sg2m1-2: clone 1–2 edited by sgRNA2 for rs12651896. Detailed DNA sequencing results for each clone are shown in Supplementary Fig 4. Vector (lentiCRISPR vector infected cells), Scr (lentiCRISPR-Scramble sgRNA infected cells), sg1nm and sg2nm (cells infected with lentiCRISPR-sgRNA1 or sgRNA2 but not detectably modified near the rs1345417 and rs12651896 sites). (I) qRT-PCR analysis of relative *SOX2* expression in heterozygously edited A375 cell clones, as above. 417-G/G (Original A375 cell line), Scr (lentiCRISPR-Scramble sgRNA infected cells), 417-G/C (the G/C-heterozygous A375 clone) Error bars represent standard errors. *p<0.05, **p<0.01, ***p<0.001, t-test comparing to vector control. N = 3 for each experiment.

To further examine whether the four variants identified in our fine mapping analyses (rs1345417, rs12651896, rs10061469 and rs1866188) play a role in the expression of nearby genes, we conducted CRISPR/Cas9-mediated gene editing for each of them. We targeted the genomic regions surrounding each variant using two different sgRNAs (S4 Fig). Our qRT-PCR analysis of annotated transcripts near these SNPs found that mixed clones of A375 cells that were stably infected with rs1345417 targeting lenti-Cas9-sgRNAs displayed significantly reduced *SOX2* expression and significantly elevated *SOX2-OT* expression compared to cells infected with control lenti-Cas9-sgRNAs (Fig 4A). Infection with rs12651896 targeting lenti-Cas9-sgRNAs led to a significant reduction in the expression of *FOXD1* (Fig 4B), while infection with rs10061469 targeting lenti-Cas9-sgRNAs showed no effect on nearby genes (Fig 4C). Infection with rs1866188 targeting lenti-Cas9-sgRNAs resulted in a significant reduction in *LIMS1* expression of (Fig 4D).

Among the genes showing significant changes in expression levels, only *SOX2* and *FOXD1* were reported to be related to hair growth [20,21]. We thus chose to focus on these two genes. For each of the two lenti-Cas9-sgRNAs targeting rs1345417 and rs12651896, we derived multiple independent single cell clones of infected A375 cells and characterized their exact deletion/mutation status at the target SNP sites (S4 Fig). We found that *SOX2* expression was significantly reduced in A375 cell clones carrying deletions/substitutions encompassing rs1345417, in comparison with cells infected with empty lenti-Cas9 vector or with lenti-Cas9-control sgRNA (Fig 4E and 4F). Importantly, A375 cell clones that were infected with rs1345417 targeting lenti-Cas9-sgRNA but where the SNP site was not successfully edited, did not show reduced *SOX2* expression (Fig 4E and 4F). Similarly, *FOXD1* expression was significantly reduced in all A375 cell clones carrying a deletion encompassing the rs12651896 site, compared to both controls and unsuccessfully edited infected cell clones (Fig 4G and 4H). In one clone (896sg1m1), β-actin expression was also unexpectedly reduced, probably due to off-target effects.

To test if a single nucleotide substitution event is sufficient to affect endogenous *SOX2* gene expression, we conducted a CRISPR/Cas9-mediated knock-in experiment at rs1345417, for which native A375 cells are homozygous (G/G). Consistent with our expectations, *SOX2* expression levels were significantly lower in an edited, G/C heterozygous A375 clone than in the original G/G A375 cells (Fig 4I).

Additionally, we conducted a luciferase reporter experiment for the genomic region surrounding rs1345417 (S5 Fig). We found that a 1,495 bp genomic fragment encompassing the rs1345417 site and containing its common G allele can act as a transcriptional enhancer. Its insertion before a *SOX2* promoter sequence led to 3 to 4-fold increase in reporter expression (S5 Fig). Moreover, a G>C mutation at rs1345417 on the reporter construct resulted in significantly reduced reporter activity, in line with our observations in the CRISPR/Cas9 experiments above (Fig 4I). Together, our data indicate that genetic variation at rs1345417 and rs12651896 can affect the transcription of *SOX2* and *FOXD1*, respectively.

### Testing for signatures of positive selection in associated regions

For some of the top associated loci, allele frequencies varied among populations (S6 Fig). This variation could be caused either by random genetic drift or by local positive selection. To assess whether positive selection could have helped shape variation in the genomic regions associated with human eyebrow thickness, we applied several statistical genetics methods. Using the Integrated Haplotype Score (iHS) [22] and the Composite of Multiple Signals (CMS) statistic [23], we did not find any evidence indicating that *SOX2*, *FOXD1* and *FOXL2* are subject to positive selection in East Asian (CHB), European (CEU) and African (YRI) populations (S7 and S8 Figs). However, there were highly significant signatures of strong positive selection in the *EDAR* region. This is not surprising, as our top signal near *EDAR* (rs1866188) is in high LD with rs3827760, a strongly selected functional SNP causing a number of ectodermal related phenotypic changes, as demonstrated previously [24]. Therefore, the signal of selection observed for rs1866188 may well be explained by the selective pressure on rs3827760. To test whether the differences in allele frequencies may stem from different local selection pressures, we applied a probabilistic approach described by He and colleagues [25] to quantify local inter-population differences in selection for the four top loci. Apart from rs1866188 at *EDAR*, we found no significant differences in selection for any other SNP in the associated regions (S9 Fig).

To test whether eyebrow thickness might be subject to polygenic selection, we applied the polygenic scores test developed by Berg and Coop [26]. This test measures the total frequency of associated alleles in a population, weighting each allele by its effect size. Loci that have undergone local positive selection will show greater divergence than expected under the neutral model. Previous studies using this method have found excess variance among populations for genetic scores associated to height, demonstrating that height is subject to local positive selection [27]. Here, we calculated the polygenic score for eyebrow thickness based on the top four associated variants. The resulting scores were similar between populations and showed no excess variance due to positive selection (*P* = 0.567, S10 Fig), indicating that the allele frequency differences observed among populations are better explained by random genetic drift than by selection. Our results thus provide no evidence that eyebrow thickness is under strong positive selection in human populations.

## Discussion

### Factors associated with eyebrow thickness

We found that eyebrow thickness is significantly associated with age and gender. As may be expected, older adults tend to have a significantly reduced eyebrow thickness due to the weakened biological function of older follicle cells. The eyebrows of males are generally thicker than those of females. Eyebrow plucking is widespread among females and may thus have affected our results; nevertheless, the large and consistent differences observed between genders are likely to be real, considering that androgens tend to stimulate hair growth [28,29].

The top associated SNP at 3q26.33, rs1345417, is located about 80 kb downstream of the *SOX2* gene (Sex Determining Region Y-Box 2), an important transcription factor that is highly expressed in the dermal condensate (DC) and dermal papilla (DP) of growing follicles [30,31]. Interestingly, fine-tuning of *SOX*2 expression appears to play an important role in the regulation of hair thickness. In murine skin development, from E18.5 onwards, Sox2 expression becomes confined to DPs in thick guard/awl/auchene hairs, but not in thin zigzag hairs [20]. Moreover, conditional ablation of Sox2 from the DP resulted in significant reduction in the length of awl/auchene, but not zigzag hairs [20]. These findings are consistent with our hypothesis that the G>C mutation at rs1345417 may cause reduced eyebrow thickness by downregulating *SOX2* expression. The top SNP within the second signal at 5q13.2, rs12651896, is located 242 kb downstream of *FOXD1* (Forkhead Box D1). This gene belongs to the forkhead family of transcription factors, whose members are characterized by a distinct forkhead domain. *FOXD1* is especially enriched in DC cells, a precursor of DP/dermal sheath niche cells within the mature follicle [21]. Finally, rs1866188 on chr2q12.3 is located 253 kb upstream of *EDAR* (Ectodysplasin A receptor), a gene which plays an important role in the development of ectodermal tissues such as hair, teeth, and sweat glands [24]. Previous studies have consistently found *EDAR* to be implicated in beard density [8] and hair thickness [24] and straightness [11,32,33].

### Regulatory variants influencing eyebrow thickness

It is notable that all of the signals identified by our GWAS fall into regulatory regions. To date, hundreds of GWAS have been conducted, resulting in the identification of a large number of genetic variants associated with common diseases and phenotypic variation. The majority (~93%) of variants associated with common traits lie within non-coding sequences, complicating their functional evaluation [34]. Recent studies have found that these variants are concentrated in regulatory DNA. This remains true even after adjusting for microarray SNP ascertainment bias and suggests that non-coding variants may act by causing changes in gene expression levels [34], as supported by several lines of evidence [35–39]. The association signals found here at 3q26.33 (rs1345417), 5q13.2 (rs12651896), and 2q12.3 (rs1866188) are all located within active enhancer regions characterized by epigenetic markers, such as H3K4me1 and H3K27ac histone modifications, and DNase hypersensitivity in epithelial cells. Our functional validation experiments provided evidence for enhancer activity around rs1345417 and rs12651896. It is thus highly plausible that these variants affect eyebrow thickness by regulating the expression of *SOX2* and *FOXD1*, respectively. In particular, the CRISPR/Cas9-mediated knock-in experiment provided direct evidence that a single substitution at rs1345417 is sufficient to affect the endogenous gene expression of *SOX2*. Our study represents a successful example of how GWAS and CRISPR/Cas9 technology can be combined to demonstrate the involvement of non-coding variants with regulatory functions in common diseases and normal phenotypic variation.

### Population heterogeneity in genes associated with eyebrow thickness

The *FOXL2* variant rs112458845 has previously been found to be associated with eyebrow thickness in Latin Americans. Here, we found no significant evidence of association of this variant with this phenotype in East Asians or Europeans. This may partly be due to the frequency distribution of the minor allele, which is rare in East Asians (4%) and absent in Europeans (0%), but relatively common in Native Americans (26%). Additionally, and perhaps more importantly, allelic effect sizes may be population specific due to a unique yet unidentified environmental or genetic background and may thus vary between Latin Americans on the one hand, and East Asians and Europeans on the other. A similar impact of population heterogeneity on allelic effect sizes has been reported for other traits, such as follicular lymphoma [40], body bone mineral density [41], and Type 1 diabetes [42].

### Eyebrow thickness is unlikely to be subject to strong positive selection

It has long been debated whether eyebrow thickness is a selected trait or a ‘neutral feature’ with no apparent link to individual fitness [3,6,7]. We used several methods to detect potential signatures of positive selection for the variants associated with eyebrow thickness. First, we tested whether any of the associated regions overlapped with signals of positive selection. We then used a recently developed probabilistic method to test and estimate differences in selection of the associated variants between populations. We further tested the presence of polygenic selection by examining subtle allele frequency shifts across multiple loci. Previous studies using the above methods have found signatures of positive selection for a range of human traits, including height, skin color, and BMI [23,25,26,43]. However, apart from the *EDAR* region around rs3827760, a SNP known to be under strong selection, we found no significant signatures of positive selection for variants associated with eyebrow thickness. These results suggest that eyebrow thickness may not be subject to strong positive selection, at least not via the genes identified here. In this context, it is worth noting that most studies postulating sexual selection of the eyebrow were based on reconstructed attractiveness [3,5,7], and it may be argued that the perception of attractiveness can vary significantly over time [3]. Therefore, while sociological studies have indicated that eyebrow thickness may be subject to sexual selection, our study does not provide any support for this conclusion from an evolutionary or genetic perspective.

In conclusion, we identified three novel genetic variants near *SOX2*, *FOXD1* and *EDAR* that influence eyebrow thickness. We demonstrated that rs1345417 and rs12651896 affect the transcriptional activity of the nearby *SOX2* and *FOXD1* genes. Furthermore, we found evidence for population heterogeneity in the genetics of eyebrow thickness. Finally, our results suggest that eyebrow thickness may not be subject to strong positive selection.

## Materials and methods

### Ethics statement

The Taizhou Longitudinal Study was carried out following protocols approved and oversight by the Institutional Research Board at Fudan University (Ethics Research Approval No.85). The Xinjiang Uyghur Study was conducted with the official approval of the Ethics Committee of the Shanghai Institutes for Biological Sciences (ER-SIBS-261410). The CANDELA ethics approval was obtained from: Universidad Nacional Autonoma de Mexico (Mexico), Universidad de Antioquia (Colombia), Universidad Peruana Cayetano Heredia (Peru), Universidad de Tarapaca (Chile), Universidade Federal do Rio Grande do Sul (Brasil) and University College London (UK, approval number 3351/001). The Rotterdam Study has been approved by the Medical Ethics Committee of the Erasmus MC (registration number MEC 02.1015) and by the Dutch Ministry of Health, Welfare and Sport (Population Screening Act WBO, license number 1071272-159521-PG). All participants provided written informed consent in these four studies.

### Populations and samples

This study is based on data from four populations (S1 Table): Han Chinese, Uyghurs, Latin Americans and Europeans. 2,961 Han Chinese (including 1,060 males and 1,901 females, with an age range of 31–87) were enrolled in Taizhou, Jiangsu Province, as part of the Taizhou Longitudinal Study (TZL) [10], in 2014. 721 Uyghurs (including 282 males and 439 females, with an age range of 17–25) were enrolled at Xinjiang Medical University, Urumqi, Xinjiang Province, China, as part of the Xinjiang Uyghur Study (UYG) in 2013–2014. Additionally, we collected summary statistics for a previously conducted GWAS on eyebrow thickness in Latin Americans of the CANDELA cohort, details of which have been previously published [8]. The Rotterdam Study is a population-based prospective study including a main cohort (RS1) and two extensions (RS2 and RS3) [12,13]. All participants were examined in detail at baseline. Collection and purification of DNA have been described in detail previously [12]. The Rotterdam Study has been entered into the Netherlands National Trial Register (NTR; www.trialregister.nl) and into the WHO International Clinical Trials Registry Platform (ICTRP; www.who.int/ictrp/network/primary/en/) under shared catalogue number NTR6831. All participants provided written informed consent to participate in the study and to have their information obtained from treating physicians.

### Ordinal phenotyping

Eyebrow thickness (i.e., density) was rated by eye on a three-point scale (TZL, UYG, RS: scarce, normal, and dense; CANDELA: low, medium and high), following an established standard and based on photographic imagery (S1 Fig). For the TZL and UYG cohorts, each case was rated independently by two different investigators. The inter-rater reliability was evaluated with the Kappa statistic. Cases where ratings were inconsistent were reviewed by a third investigator, who made the final decision. For the CANDELA cohort, an interview of the volunteers had indicated that most women modified their eyebrows; in this cohort, eyebrow thickness was therefore only scored in men. Phenotyping was performed by an experienced investigator, and intra-rater reliability was assessed for 150 individuals. For RS, three investigators simultaneously and independently evaluated all photos, which were displayed on two identical screens with the same settings. Before evaluation, a total of 50 photos were openly discussed to reach a consensus between the three investigators. Inter-rater reliability was also evaluated with the Kappa statistic. The average score from the three independent ratings was used as the final phenotype in all subsequent analysis.

### Genotype quality control and imputation

For TZL and UYG, blood samples were collected, and DNA was extracted. All samples were genotyped using the Illumina HumanOmniZhongHua-8 chip, which interrogates 894,517 SNPs. To control for genotype quality, we used PLINK 1.9 [44] to exclude individuals with more than 5% missing data, related individuals, and those that failed the X-chromosome sex concordance check, or for whom the available information on ethnicity was incompatible with their genetic information. We also excluded SNPs with more than 2% missing data, with a minor allele frequency (MAF) below 1%, and those that failed the test for Hardy-Weinberg equilibrium (*P*<1×10^−5^). The chip genotype data were phased using SHAPEIT [45], and IMPUTE2 [46] was then used to impute genotypes at non-genotyped SNPs using variant positions from the 1000 Genomes Phase 3 data as a reference. SNPs with an imputation quality score (INFO) below 0.8 or a MAF below 1% were eliminated from further analyses. For the Han Chinese population, a total of 6,343,243 imputed SNPs passed quality control and were combined with 776,213 genotyped SNPs for association analysis. For the Uyghur population, a total of 6,414,304 imputed SNPs passed quality control and were combined with 810,648 genotyped SNPs for further analyses. In RS1 and RS2, genotyping was carried out using the Infimum II HumanHap550K Genotyping Bead Chip version 3, which contains 6,787,905 probes. Complete information on genotyping protocols and quality control measures for RS1 and RS2 have been described previously [47,48]. In RS3, genotyping methods closely followed those established for RS1 and RS2, but a denser array, the Human 610 Quad Arrays of Illumina with 15,880,747 probes, was used. Individuals with a call rate < 97.5%, gender mismatch with typed X-linked markers, or excess autosomal heterozygosity (>0.33) were excluded, as were duplicates or 1st degree relatives identified using IBS probabilities, and outliers using multi-dimensional scaling analysis with reference to the 210 Hap Map samples. Genome-wide imputation in RS3 closely followed the methods used in RS1 and RS2, as described in detail previously [48]. Genotypes were imputed using MACH [49] based on phased autosomal chromosomes of the 1000 Genome reference panel, orientated on the positive strand.

### Population stratification analysis

The effects of possible population stratification were corrected using the EIGENSTRAT [50] tool from the EIGENSOFT package. To this end, TZL and UYG data were combined with 1000 Genomes Phase 3 data for YRI, CHB and CEU populations. 102,284 SNPs in low linkage equilibrium (r^2^<0.2) were selected for analysis. Principal component (PC) analysis did not find any outliers in TZL and UYG (S11 Fig).

### Statistical genetics analyses

Initial genome-wide association tests using multiple linear regression with an additive genetic model incorporating gender, age and four genetic PCs as covariates were performed in PLINK 1.9 [44]. Expected and observed association results for all tests were visualized in quantile-quantile (Q-Q) plots to assess systematic inflation in association resulting from population stratification or other systematic causes of bias. None of the Q-Q plots showed any sign of inflation, the genomic control factor λ being < 1.06 in all cases (S3 Fig). To evaluate the presence of additional independent signals at each locus, we performed conditional analyses by adding the dosages of the top SNP at each locus to the regression model. Q-Q, Manhattan and regional association plots [51] were created in R. The proportion of variance in eyebrow thickness explained by the genetic variants identified was estimated using GCTA [52]. The meta-analysis of the TZL, UYG and CANDELA data sets was performed using METAL [53]. Heterogeneity of SNP associations across studies was tested via Cochran’s Q statistic [54], and its magnitude was expressed by I^2^. For SNPs with significant heterogeneity, a random effects model was applied for meta-analysis using METASOFT [55].

### Fine mapping and credible set construction

We performed fine mapping of each locus for a 1 Mb genomic interval flanking the top SNP (500 kb upstream and 500 kb downstream) using PAINTOR [14,15]. For each SNP within this 1 Mb region, the posterior probability that this SNP is driving the region’s association signal was calculated by dividing the SNP’s Bayes factor (BF) by the sum of the BFs of all SNPs in the region [56]. A 99% credible set was then constructed by (1) ranking all variants according to their Bayes factor, and (2) including ranked variants until their cumulative posterior probability of representing the causal variant at a given locus exceeded 0.99 [57].

### Functional annotation

To further facilitate the prioritization of variants for functional analysis, we used CADD [16] (Combined Annotation-Dependent Depletion) and DeepSEA [17] (deep learning-based sequence analyzer) to evaluate the possible functional consequences of the variants in the 99% credible set.

### Candidate gene identification and regulatory annotation

Candidate genes were chosen based on their distance to the associated loci as well as their function, involvement in biochemical pathways, tissue expression, and involvement in similar phenotypes. The relevant information was obtained from NCBI [58] and Ensemble [59], as well as available published data. We used HaploReg v4.1 [60] to extract a variety of regulatory annotations, including histone modification (ChIP-seq tracks), chromatin state segmentations (15-state) and ChIA-PET [61] (Chromatin Interaction) from ENCODE [18] and the Roadmap Epigenomics Project [19], conserved regions from GERP [62] and Phastcons [63], and eQTLs from the GTEx [64] and GEUVADIS databases [65].

### Cell culture

No cell lines were found in the database of commonly misidentified cell lines maintained by ICLAC and NCBI Biosample. Cell lines were not authenticated. NHEM (human melanocytes) and A375 (human melanoma) were purchased from the cell bank of the Chinese Academy of Sciences. HACAT (immortal keratinocytes) and SCC13 (skin squamous-cell carcinoma cell line) were purchased from ATCC. Cell lines were routinely tested for mycoplasma infection. Cells were cultured using DMEM+10%FBS+1%Penicillin-Streptomycin.

### Plasmid construction and luciferase assays

A 1,495 bp genomic fragment comprising the rs1345417 enhancer was amplified from human genomic DNA and cloned into a pGL3-promoter vector, in which the SV40 promoter was replaced by a *SOX2* promoter. The G>C mutation was introduced by site-directed mutagenesis (Takara). The inserts in each construct were verified by sequencing. The detail information of primer sequences can be found in S5 Table. Constructs were transfected with equimolar amounts (500 ng) of luciferase reporter plasmids into A375 Melanoma cells using jetPEI (Polyplus), according to the manufacturer’s instructions. Luciferase expression was normalized to 200 ng Renilla luciferase expression (pRL-SV40). Cells were harvested after 48 h. Luminescence activity was measured with a Berthold Centro LB 960 Microplate Luminometer. Data represent at least three independent experiments. Student’s two-tailed t-test was used to determine statistical significance.

### CRISPR/Cas9-mediated gene editing

For each candidate variant, two sgRNAs were designed, cloned into the lentiCRISPR v2 vector, and packaged into lentivirus as previously described [66]. sgRNAs used for rs1345417: sgRNA1 (CCTGCTTTTGCCTCAGCCCACAT) and sgRNA2 (CCCACATCTTCTCTATTAGTAAG). sgRNAs used for rs12651896: sgRNA1 (CAAAATGTTCTTGCTAGCATATCCA) and sgRNA2 (GCATATCCATAACTAGCACAGG). sgRNAs used for rs10061469: sgRNA1 (ACAACCTGCAATAAACTATTAA). sgRNAs for rs1866188 site: sgRNA1 (GAGTGGCCACTCTCTTTTGC) and sgRNA2 (GAATGCATAAGGA TCAAATCG). Scramble Control sgRNA sequence is (CCCACATAGTCTCACTTAG TAAG).

For target site deletion via lentiCRISPR-sgRNA virus infection, stably infected cells were selected on puromycin. For clone selection, stably infected cells were diluted to allow colonial growth. Single colonies were individually picked for DNA sequencing of the target site. Because protospacer adjacent motives were located as far as 100 bp upstream and 22 bp downstream, the deletion at rs1866188 was considerably larger than for the other sites.

For G>C substitution at rs1345417 in A375 cells, we used a CRISPR-Cas9 mediated knock-in strategy. Specifically, in order to substitute the G/G of rs1345417 in A375 cell, a ~1000bp DNA fragment encompassing the rs1345417 site from a Chinese individual with a C/C homozygote genotype was PCR amplified and TA cloned into the pMD18T Vector (Takara) to construct a C-donor plasmid. We then co-transfected the C-donor plasmid and the lenticrispr-rs1345417-sgRNA2 plasmid into A375 cells using the Effectene reagent (Qiagen). 48 h after transfection, the cells were selected on puromycin for 48 h to eliminate untransfected cells. Successfully transfected cells were then diluted for colonial growth. Single colonies were individually picked for DNA sequencing to screen for substitution at the target site. In total, 44 clones were screened.

### Molecular biology

Sequencing of the rs1345417 site: Genomic DNA was extracted from each cell clone using ZR Genomic DNA-tissue MiniPrep (Zymo) following the manufacturer’s protocol. The rs1345417 genomic region was amplified from genomic DNA by nested PCR and TA-cloned into pMD18 T vector for sequencing. At least four individual TA-clones were sequenced for each cell clone. RNA extraction was conducted using Direct-zol RNA MiniPrep Plus (Zymo) following the manufacturer’s protocol. Reverse transcription was conducted using the iscript cDNA synthesis kit (Biorad) following the manufacturer’s protocol. Real-time PCR experiments were conducted on a VIIA7 Fast Real-Time PCR system (Applied Biosystem) using the iTaq universal SYBR Green supermix (Biorad). All statistical analyses were conducted using Microsoft Excel and the GraphPad Prism 6 software.

### Tests for positive selection

Genomic characteristics resulting from strong recent positive selection include low haplotype diversity and high linkage disequilibrium. We calculated extended haplotype homozygosity (EHH) [22,67] for all SNPs until EHH < 0.05 in CEU, CHB and UYG samples. Next, the integrated haplotype score (iHS) [68] was calculated for all SNPs, with an allele frequency bin of 0.05 to standardize iHS scores against other SNPs of the same frequency class within the region. Finally, we calculated P values assuming a Gaussian distribution of iHS scores under the neutral model, which was checked by plotting the values against a Gaussian distribution. The empirical significance cutoff was based on the top 0.1% iHS scores. We also performed genome-wide CMS [23] analysis in African (YRI), European (CEU), and East Asian (JPT+CHB) populations from the 1000 Genome Project [69] to validate our results. The empirical significance cutoff was based on the top 0.1% CMS scores. Differences in allele frequencies are indicators of possible differences in selection between populations. To test whether the differences in allele frequencies may result from different local selection pressure, we used a probabilistic method which was recently put forward by He and colleagues [25]. We used this approach to test and estimate selection differences between populations for the four top associated loci. We first estimated differences in selection coefficients between populations (CHB, CEU and YRI) using logarithmic odds ratios of allele frequencies. The variance of the estimation was then calculated based on genome-wide variants. Finally, we calculated P values assuming a Gaussian distribution of the statistic under the neutral model. The significance cutoff was P<0.005 after multiple testing correlation.

To investigate whether the loci associated with eyebrow thickness are more differentiated among populations than expected under neutral genetic drift, for each population *m*, we calculated the polygenic eyebrow thickness score (genetic score) as

$$Z_{m}=2\sum_{i=1}^{L}\beta_{l}p_{ml}$$

where *β*_*l*_ is the effect size of the eyebrow thickness increasing allele *l*, and *p*_*ml*_ is the frequency of allele *l* in population *m*. We first used the four loci identified here in conjunction with allele frequency data from the 1000 Genome Project dataset to estimate the genetic score for eyebrow thickness in each population, with the effect sizes estimated in the meta-analysis. To test whether there was a signature of polygenic adaptation, we then adopted a framework developed by Berg and Coop [26], which builds a multivariate normal model based on matched, presumably neutral variants, to account for relationships among populations. Traits that have undergone local selection will show excess divergence among populations (significance cutoff: P<0.05).

## Supporting information

S1 Fig Representative images of eyebrow thickness. Level 1 (scarce): low eyebrow density, the brow does not cover the skin completely. Level 2 (normal): the eyebrow covers the skin, there is no hair between the two brows. Level 3 (dense): high eyebrow density, the color of the eyebrow is darker than for level 2.
(PNG)

S2 Fig Manhattan plot showing the results of the GWAS for eyebrow thickness in the Uyghurs. Manhattan plot illustrating the results of the genome-wide scan for eyebrow thickness in 721 Uyghurs after adjusting for the top four PCs, gender and age. The red line indicates the threshold for genome-wide statistical significance (*P*<5×10^−8^).
(PNG)

S3 Fig Quantile-Quantile (Q-Q) plots for the four GWAS included in this study. a) TZL, b) UYG, c) CANDELA and d) meta-analysis.
(PNG)

S4 Fig CRISPR/Cas9-mediated deletion in the human A375 cell line. DNA sequences of the regions around a) rs1345417, b) rs12651896, c) rs10061469 and d) rs1866188 in original A375 cells and their CRISPR-Cas9 edited clones. For rs1345417, 417sg1m1-2: clone 1 and 2 edited by sgRNA1. 417sg2m1-3: clone 1–3 edited by sgRNA2. 417-G/C: a single nucleotide substitution event. For rs12651896, 896sg1m1-2: clone 1 and 2 edited by sgRNA1. 896sg2m1-2: clone 1–2 edited by sgRNA2. For rs1866188, clone 1 and 2 edited by both sgRNA1 and sgRNA2.
(PNG)

S5 Fig Luciferase reporter assays linking rs1345417 to altered levels of gene expression. A) Schematic diagram of the constructed luciferase reporters. Triangles and green rectangles represent the promoter and luciferase gene, respectively. The putative regulatory element (1,495-bp insertion) is indicated by yellow rectangles, with the SNP rs1345417 represented as a red line (G>C). pGL3-Basic, empty vector (SV40 promoter); SOX2-Pro, vector containing the SOX2 promoter; SOX2-G, G allele at rs1345417; SOX2-C, C allele at rs1345417. B-D) Three repeats of luciferase reporters in human A375 cells. Bars represent the mean intensity of luciferase gene activity relative to the empty vector (pGL3-Basic) as a control, measured 48h after transfection for a typical experiment. Error bars represent standard errors. *p<0.05, **p<0.01, ***p<0.001, t-test comparing to vector control. N = 3 for each experiment.
(PNG)

S6 Fig Geographical distribution of the minor allele frequencies for the significant variants identified here, based on the Human Genome Diversity Project. Allele frequency data from 53 world-wide populations were taken from the Human Genome Diversity Project. For a) rs1345417, b) rs12651896 and c) rs1866188, ancestral alleles are represented in blue, derived alleles in orange.
(PNG)

S7 Fig Results of the selection analysis on EDAR, *FOXL2*, *SOX2* and *FOXD1* based on CMS scores. CMS scores are plotted against physical distance for a) *EDAR*, b) *FOXL2*, c) *SOX2* and d) *FOXD1* regions in CEU, CHB+JPT and YRI populations. The yellow line marks the location of the index SNP in each region (Table 1). The black dashed line indicates the threshold of top 0.1% chromosome-wide CMS scores (>4.8).
(PNG)

S8 Fig Results of the selection analysis on *EDAR*, *FOXL2*, *SOX2* and *FOXD1* based on iHS scores. Absolute iHS scores are plotted against physical distance for a) *EDAR*, b) *FOXL2*, c) *SOX2* and d) *FOXD1* regions in CEU, CHB+JPT and YRI populations. The yellow line marks the location of the index SNP in each region (Table 1). The black dashed line indicates the threshold of top 0.1% chromosome-wide iHS scores (>3.1).
(PNG)

S9 Fig Differences in selection coefficients between populations. The forest plot illustrates the differences in selection coefficients of variants in four independent genomic signals for eyebrow thickness. Differences are shown between three 1000 Genomes populations (CEU, CHB, YRI). For each variant, points (and error bars) indicate the estimated differences in selection coefficients (and 99.5% confidence intervals; after multiple testing). Each population pair is denoted by a different color. The underlying data were obtained from phase 3 of the 1000 Genomes Project.
(PNG)

S10 Fig Visual representation of polygenic selection for eyebrow thickness. The genetic score was calculated on the basis of the four associated variants, with effect sizes estimated in the meta-analysis and allele frequencies obtained from phase 3 of the 1000 Genomes Project.
(PNG)

S11 Fig Principle component analysis of individuals from Han Chinese, Uyghur and 1000 Genome Project samples. Principal component analysis of 102,284 SNPs (r2<0.2) in Han Chinese (n = 2961), Uyghurs (n = 721), and three 1000 Genome population samples (97 CHB, 86 CEU and 88 YRI) placed Han Chinese and Uyghurs into distinct clusters. There were no significant population outliers in our samples.
(PNG)

S1 Table Sample phenotype information. (DOCX)

S2 Table Phenotyping concordance between cohorts. (DOCX)

S3 Table Meta-analysis results for index SNPs. (DOCX)

S4 Table Posterior probability and functional annotation of variants at associated loci. (DOCX)

S5 Table Primer sequences for plasmid construction and luciferase assays. (DOCX)
