# Supplementary figures and images for: Genome-wide association studies and CRISPR/Cas9-mediated gene editing identify regulatory variants influencing eyebrow thickness in humans
Source: PLoS Genet. 2018 Sep 24;14(9):e1007640. doi: 10.1371/journal.pgen.1007640 (PMC6171961; doi:10.1371/journal.pgen.1007640)

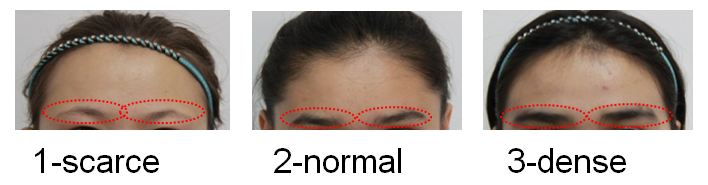

Supplement: S1 Fig — Level 1 (scarce): low eyebrow density, the brow does not cover the skin completely. Level 2 (normal): the eyebrow covers the skin, there is no hair between the two brows. Level 3 (dense): high eyebrow density, the color of the eyebrow is darker than for level 2. (PNG) [file pgen.1007640.s001.png]

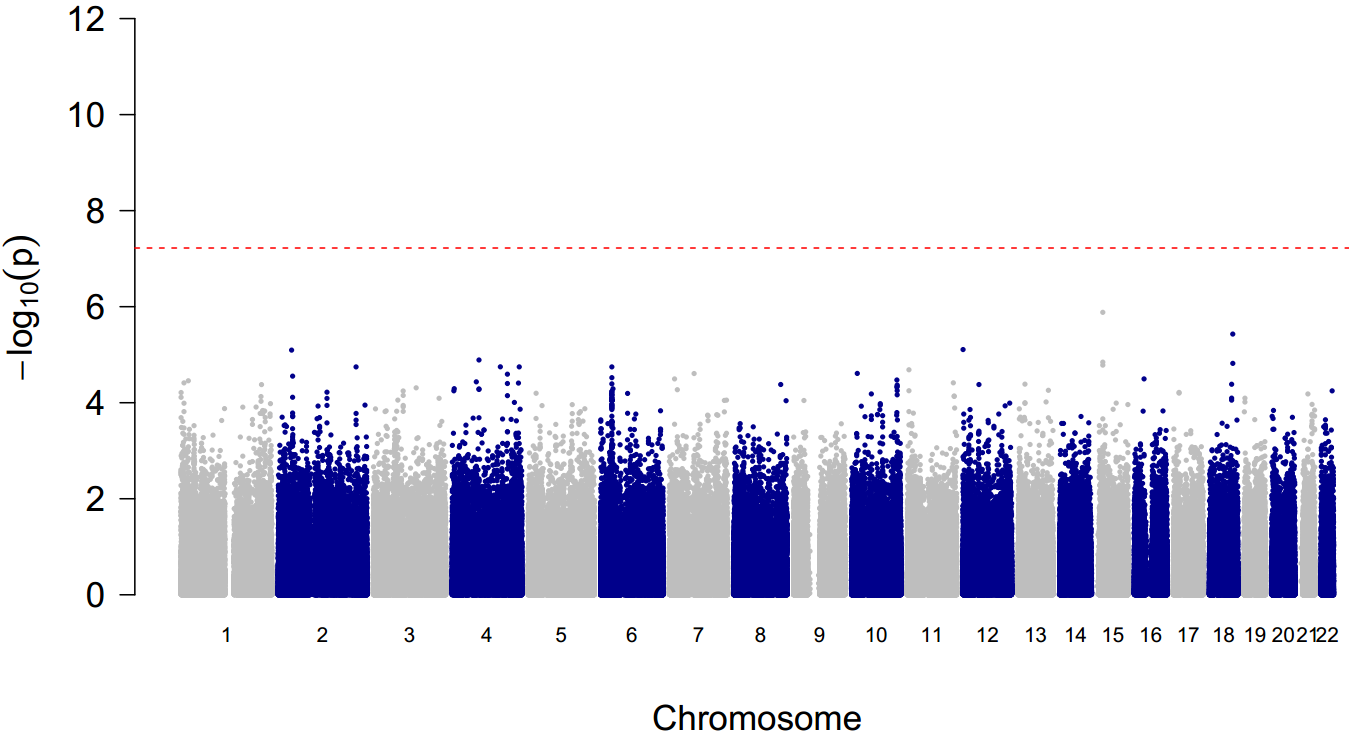

Supplement: S2 Fig — Manhattan plot illustrating the results of the genome-wide scan for eyebrow thickness in 721 Uyghurs after adjusting for the top four PCs, gender and age. The red line indicates the threshold for genome-wide statistical significance (P<5×10^−8^). (PNG) [file pgen.1007640.s002.png]

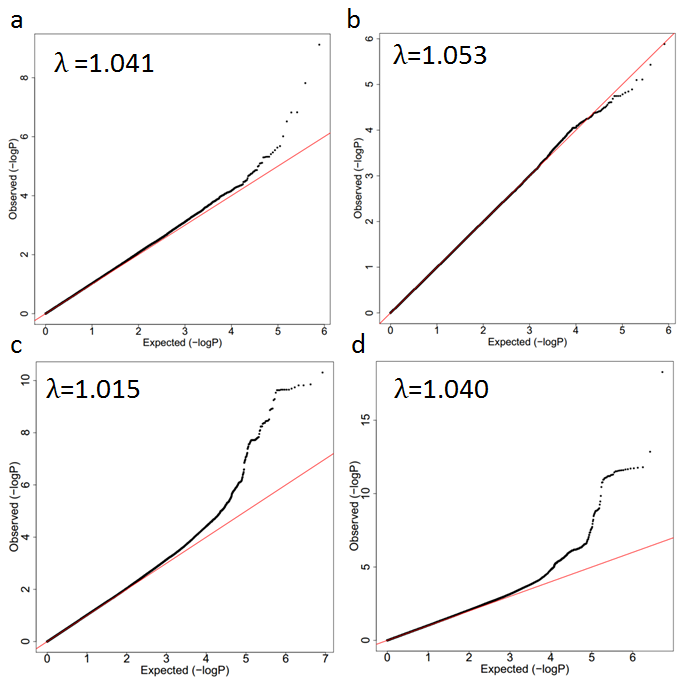

Supplement: S3 Fig — a) TZL, b) UYG, c) CANDELA and d) meta-analysis. (PNG) [file pgen.1007640.s003.png]

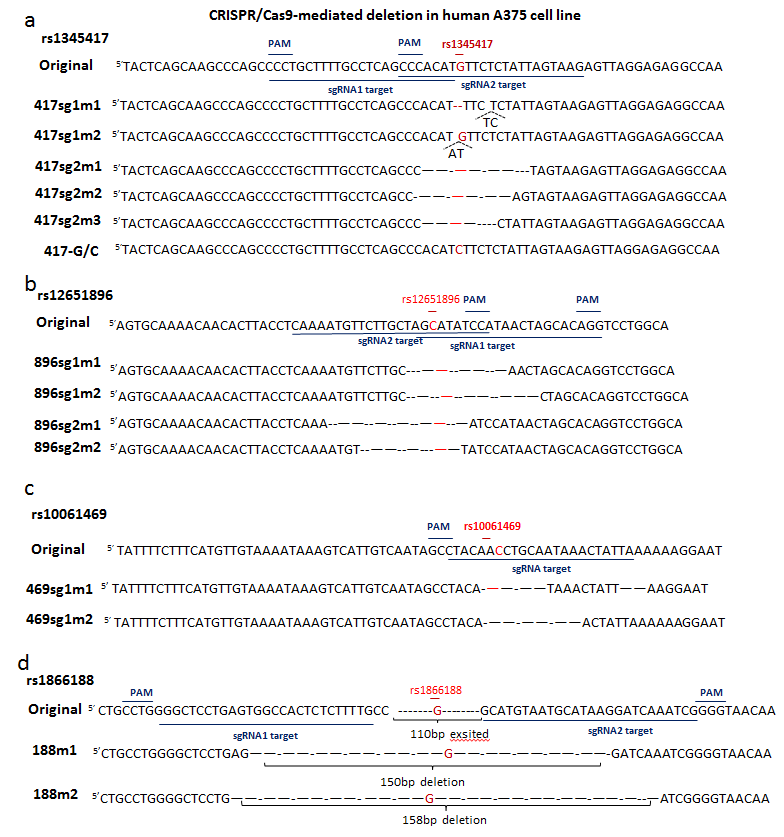

Supplement: S4 Fig — DNA sequences of the regions around a) rs1345417, b) rs12651896, c) rs10061469 and d) rs1866188 in original A375 cells and their CRISPR-Cas9 edited clones. For rs1345417, 417sg1m1-2: clone 1 and 2 edited by sgRNA1. 417sg2m1-3: clone 1–3 edited by sgRNA2. 417-G/C: a single nucleotide substitution event. For rs12651896, 896sg1m1-2: clone 1 and 2 edited by sgRNA1. 896sg2m1-2: clone 1–2 edited by sgRNA2. For rs1866188, clone 1 and 2 edited by both sgRNA1 and sgRNA2. (PNG) [file pgen.1007640.s004.png]

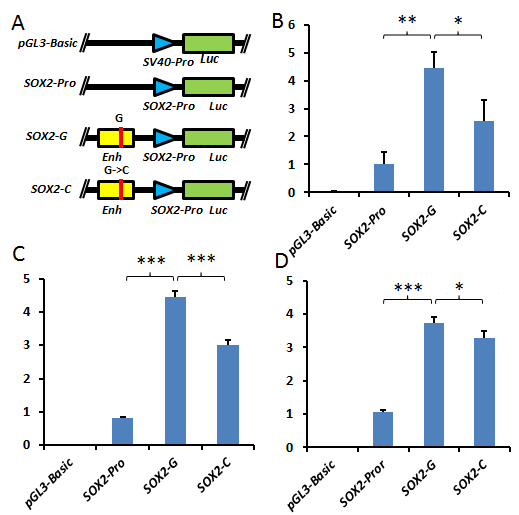

Supplement: S5 Fig — A) Schematic diagram of the constructed luciferase reporters. Triangles and green rectangles represent the promoter and luciferase gene, respectively. The putative regulatory element (1,495-bp insertion) is indicated by yellow rectangles, with the SNP rs1345417 represented as a red line (G>C). pGL3-Basic, empty vector (SV40 promoter); SOX2-Pro, vector containing the SOX2 promoter; SOX2-G, G allele at rs1345417; SOX2-C, C allele at rs1345417. B-D) Three repeats of luciferase reporters in human A375 cells. Bars represent the mean intensity of luciferase gene activity relative to the empty vector (pGL3-Basic) as a control, measured 48h after transfection for a typical experiment. Error bars represent standard errors. *p<0.05, **p<0.01, ***p<0.001, t-test comparing to vector control. N = 3 for each experiment. (PNG) [file pgen.1007640.s005.png]

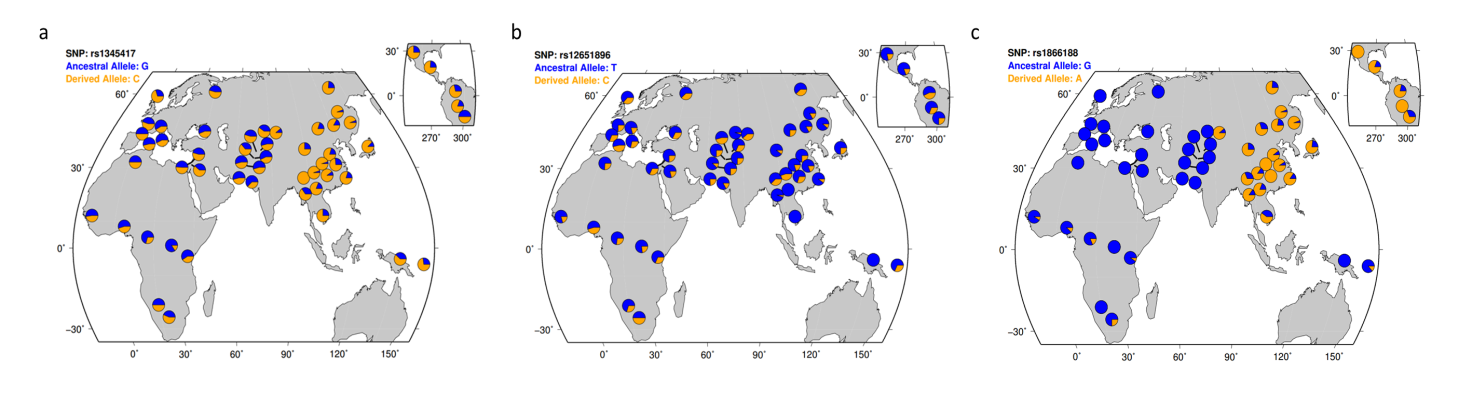

Supplement: S6 Fig — Allele frequency data from 53 world-wide populations were taken from the Human Genome Diversity Project. For a) rs1345417, b) rs12651896 and c) rs1866188, ancestral alleles are represented in blue, derived alleles in orange. (PNG) [file pgen.1007640.s006.png]

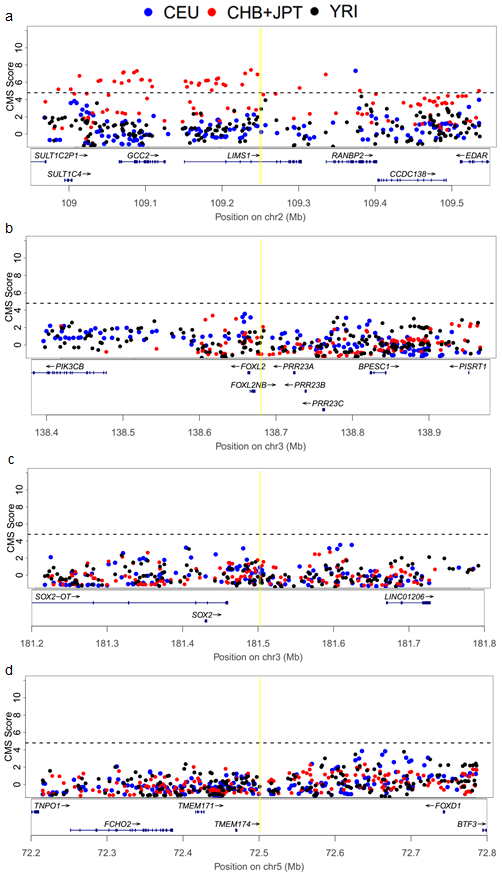

Supplement: S7 Fig — CMS scores are plotted against physical distance for a) EDAR, b) FOXL2, c) SOX2 and d) FOXD1 regions in CEU, CHB+JPT and YRI populations. The yellow line marks the location of the index SNP in each region (Table 1). The black dashed line indicates the threshold of top 0.1% chromosome-wide CMS scores (>4.8). (PNG) [file pgen.1007640.s007.png]

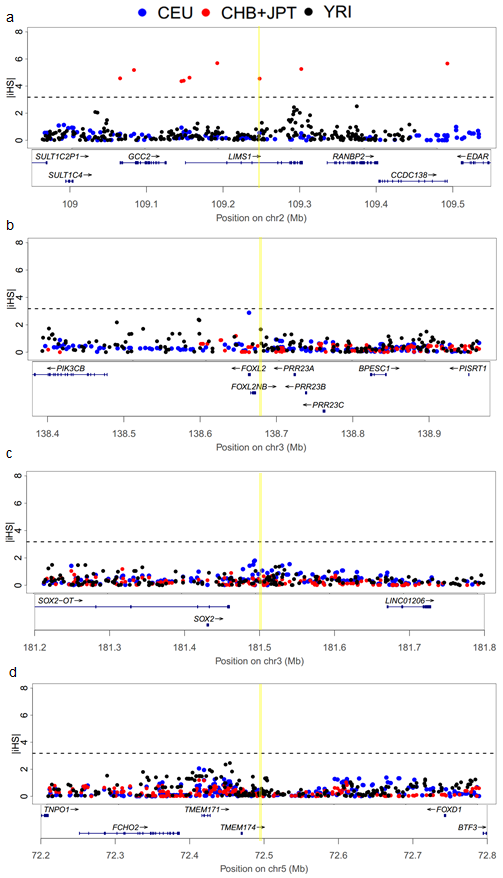

Supplement: S8 Fig — Absolute iHS scores are plotted against physical distance for a) EDAR, b) FOXL2, c) SOX2 and d) FOXD1 regions in CEU, CHB+JPT and YRI populations. The yellow line marks the location of the index SNP in each region (Table 1). The black dashed line indicates the threshold of top 0.1% chromosome-wide iHS scores (>3.1). (PNG) [file pgen.1007640.s008.png]

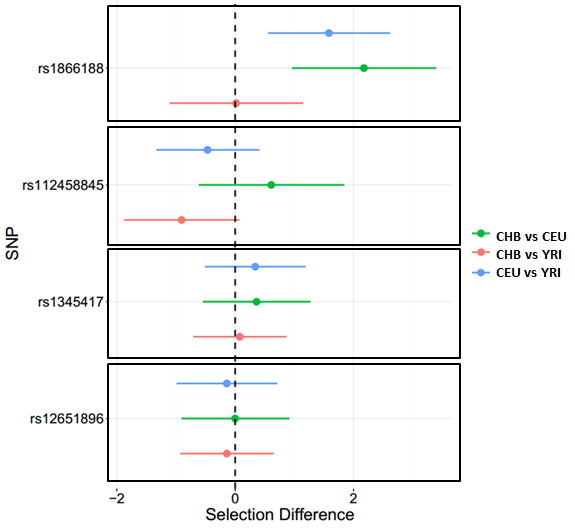

Supplement: S9 Fig — The forest plot illustrates the differences in selection coefficients of variants in four independent genomic signals for eyebrow thickness. Differences are shown between three 1000 Genomes populations (CEU, CHB, YRI). For each variant, points (and error bars) indicate the estimated differences in selection coefficients (and 99.5% confidence intervals; after multiple testing). Each population pair is denoted by a different color. The underlying data were obtained from phase 3 of the 1000 Genomes Project. (PNG) [file pgen.1007640.s009.png]

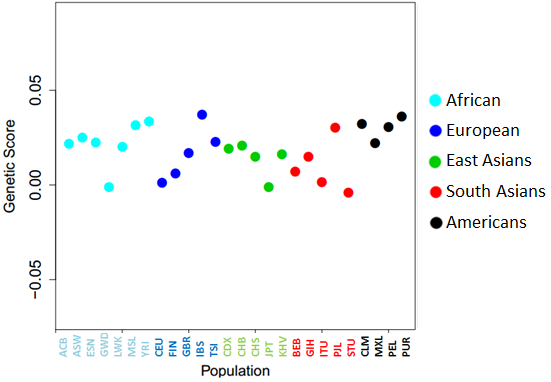

Supplement: S10 Fig — The genetic score was calculated on the basis of the four associated variants, with effect sizes estimated in the meta-analysis and allele frequencies obtained from phase 3 of the 1000 Genomes Project. (PNG) [file pgen.1007640.s010.png]

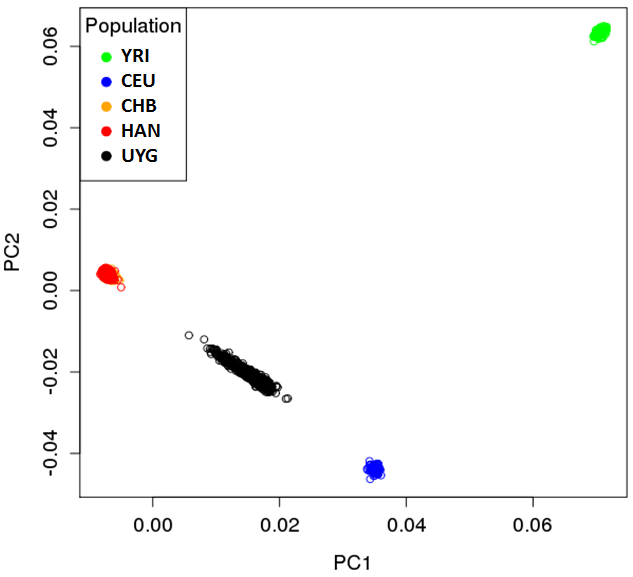

Supplement: S11 Fig — Principal component analysis of 102,284 SNPs (r2<0.2) in Han Chinese (n = 2961), Uyghurs (n = 721), and three 1000 Genome population samples (97 CHB, 86 CEU and 88 YRI) placed Han Chinese and Uyghurs into distinct clusters. There were no significant population outliers in our samples. (PNG) [file pgen.1007640.s011.png]
