# Supplementary material for: Genome-wide association studies and CRISPR/Cas9-mediated gene editing identify regulatory variants influencing eyebrow thickness in humans
Source: PLoS Genet. 2018 Sep 24;14(9):e1007640. doi: 10.1371/journal.pgen.1007640 (PMC6171961; doi:10.1371/journal.pgen.1007640)
Supplement: S1 Table — (DOCX) [file pgen.1007640.s012.docx]

**S1 Table. Sample phenotype information.**

| Cohort | Scarce | Normal | Dense | Gini-index |
| --- | --- | --- | --- | --- |
| TZL (discovery)* | 790 | 1968 | 203 | 0.52 |
| UYG (replication)* | 68 | 335 | 318 | 0.58 |
| CANDELA (replication)* | 168 | 601 | 1532 | 0.49 |
| RS (replication)** | 2133 | 1905 | 373 | 0.57 |

*Data from TZL, UYG, and CADELA were used for meta-analysis. **Data from RS were only used for replication.
