# Supplementary material for: Genome-wide association studies and CRISPR/Cas9-mediated gene editing identify regulatory variants influencing eyebrow thickness in humans
Source: PLoS Genet. 2018 Sep 24;14(9):e1007640. doi: 10.1371/journal.pgen.1007640 (PMC6171961; doi:10.1371/journal.pgen.1007640)
Supplement: S2 Table — (DOCX) [file pgen.1007640.s013.docx]

**S2 Table.** **Phenotyping concordance between cohorts**

| Cohort | TZL^a^ | UYG^a^ | CANDELA^b^ | RS^a^ |
| --- | --- | --- | --- | --- |
| Kappa | 0.59 | 0.65 | 0.59 | 0.48, 0.66, 0.49 |

^a^Inter-rater reliability. For RS, three researchers independently evaluated all photos.

^b^Intra-rater reliability.
