# Supplementary material for: Genome-wide association studies and CRISPR/Cas9-mediated gene editing identify regulatory variants influencing eyebrow thickness in humans
Source: PLoS Genet. 2018 Sep 24;14(9):e1007640. doi: 10.1371/journal.pgen.1007640 (PMC6171961; doi:10.1371/journal.pgen.1007640)
Supplement: S3 Table — (DOCX) [file pgen.1007640.s014.docx]

**S3 Table. Meta-analysis results for index SNPs.**

| SNP | P value  (F.E. model)^a^ | P value  (R.E. model)^b^ | Cochran’s Q | P value  (Cochran’s Q) | I^2 c^ |
| --- | --- | --- | --- | --- | --- |
| rs1866188 | 3.54×10^-11^ | 5.81×10^-11^ | 0.301 | 0.960 | 0 |
| rs112458845 | 8.85×10^-8^ | 2.24×10^-9^ | 16.28 | 9.92×10^-4^ | 81.58% |
| rs1345417 | 5.20×10^-19^ | 1.11×10^-19^ | 2.14 | 0.544 | 0 |
| rs12651896 | 1.40×10^-13^ | 2.52×10^-13^ | 1.21 | 0.750 | 0 |

^a^ F.E. model, fixed effect model.

^b^ R.E. model, random effect model.

^c^I^2^ heterogeneity index.
