# Supplementary material for: Genome-wide association studies and CRISPR/Cas9-mediated gene editing identify regulatory variants influencing eyebrow thickness in humans
Source: PLoS Genet. 2018 Sep 24;14(9):e1007640. doi: 10.1371/journal.pgen.1007640 (PMC6171961; doi:10.1371/journal.pgen.1007640)
Supplement: S4 Table — (DOCX) [file pgen.1007640.s015.docx]

**S4 Table. Posterior probability and functional annotation of variants at associated loci**

| **Signal** | **rsID** | **P value**  **(F.E.)** ^a^ | **Posterior probability** | **Cumulative probability** | **CADD** | **DeepSEA** |
| --- | --- | --- | --- | --- | --- | --- |
| 2q12.3 | **rs1866188**^b^ | 3.54×10^-11^ | 0.999 | 0.999 | 2.830 | 2.721 |
| 3q26.33 | **rs1345417**^b^ | 5.20×10^-19^ | 1 | 1 | 19.290 | 11.609 |
| 5q13.2 | **rs12651896**^b^ | 1.40×10^-13^ | 0.744 | 0.744 | 4.206 | 2.326 |
|  | **rs10061469**^b^ | 3.34×10^-12^ | 0.084 | 0.828 | 19.4 | 10.378 |
|  | rs9654421 | 1.72×10^-12^ | 0.067 | 0.895 | 0.277 | 1.977 |
|  | rs6870798 | 3.57×10^-12^ | 0.051 | 0.946 | 2.539 | 2.577 |
|  | rs10074446 | 8.00×10^-12^ | 0.015 | 0.961 | 2.173 | 3.098 |
|  | rs34804116 | 2.78×10^-12^ | 0.012 | 0.974 | 2.948 | 2.686 |
|  | rs10473935 | 4.98×10^-12^ | 0.007 | 0.980 | 0.028 | 1.143 |
|  | rs9654420 | 3.05×10^-12^ | 0.006 | 0.986 | 0.641 | 3.428 |
|  | rs6882248 | 6.38×10^-12^ | 0.006 | 0.992 | 4.206 | 2.326 |

^a^ F.E., fixed effect model.

^b^ prioritized variants considering the posterior probability and functional annotation.
