# Supplementary material for: Genome-wide association studies and CRISPR/Cas9-mediated gene editing identify regulatory variants influencing eyebrow thickness in humans
Source: PLoS Genet. 2018 Sep 24;14(9):e1007640. doi: 10.1371/journal.pgen.1007640 (PMC6171961; doi:10.1371/journal.pgen.1007640)
Supplement: S5 Table — (DOCX) [file pgen.1007640.s016.docx]

**S5 Table. Primer sequences for Plasmid construction and luciferase assays**

| **Primer** | **Sequence (5´-3´)** |
| --- | --- |
| *SOX2* promoter For | GGAAGATCTCGACCCCGCCTCCCAG |
| *SOX2* promoter Rev | CCCAAGCTTAGAATAATTTGGGGGAAAAAAAG |
| Enhancer For | CGGGGTACCAGATCTGTAGCACCTGGGTCTAC |
| Enhancer Rev | CTAGCTAGCGTCGACTTTTAGAGTTAAAAAACAACCC |
| rs1345417 For | GCCCACATGTTCTCTATTAGTAAGAG |
| rs1345417 Rev | CTCTTACTAATAGAGAACATGTGGGC |
